# Editorial: Early palliative care for cancer patients

**DOI:** 10.3389/fonc.2023.1207587

**Published:** 2023-06-22

**Authors:** Marco Maltoni, Augusto Caraceni, Pal Klepstad, Romina Rossi

**Affiliations:** ^1^ Medical Oncology Unit, Department of Medical and Surgical Sciences (DIMEC), University of Bologna, Bologna, Italy; ^2^ Palliative care, Pain Therapy and Rehabilitation Unit, Fondazione IRCCS Istituto Nazionale dei Tumori, Milan, Italy; ^3^ Dipartimento di Scienze Cliniche e di Comunità, Università degli Studi di Milano, Milan, Italy; ^4^ Department of Circulation and Medical Imaging, Norwegian University of Science and Technology (NTNU), Trondheim, Norway; ^5^ Department of Anesthesiology and Intensive Care Medicine, St Olavs Hospital, Trondheim University Hospital, Trondheim, Norway; ^6^ Palliative Care Unit, IRCCS Istituto Romagnolo per lo Studio dei Tumori (IRST) "Dino Amadori", Meldola, Italy

**Keywords:** early palliative care, end of life care, multidisciplinary approach, advanced cancer patients, patients needs

In the past, oncological palliative care (PC) had been identified as “end-of-life palliative care” (EoLPC), and EoLPC always began by carrying out a prognostic evaluation of life expectancy when antitumor therapies had been exhausted. In recent years, however, this approach has been modified, giving way to a process of PC based on needs, not only at the end of life, and not only after having withdrawn all antineoplastic treatments. This non-EoLPC process has been variously identified as early palliative care (EPC), simultaneous palliative care (SPC), or timely palliative care (TPC) (1–3). EPC is generally delivered together with anticancer therapies, while EoLPC represents the unique treatment delivered at that end of the disease trajectory.

From an organizational point of view, EPC is mainly provided in outpatient and consultant settings within acute hospitals, while EoLPC is mainly in home-care programs or hospice residential facilities (4). The best methods of activation of palliative care and referral to the palliative care team by oncologists are still the subject of studies and clinical research (5). Timely provision of seamless palliative care across all settings must be guaranteed without interruptions (6).

EPC has among its aims a favorable impact on the quality of life, on the quality of care, and finally on costs (7). In fact, in addition to objectives related to individual patient benefits, a personalization of treatment can allow a more appropriate use of health care services and an impact on indicators of therapeutic aggressiveness at the end of life: timelier referral to end-of-life palliative care and reduction of futility of treatments (8).

Clinical trials and systematic reviews have highlighted, among the outcomes on which efficacy has been demonstrated, quality of life, symptom burden, satisfaction, communication, caregivers outcomes, EoL care, and, with more controversial evidence, survival (9, 10).

In some cases, systematically activated EPC did not have a clinically or statistically favorable impact. Reasons for possible lack of effectiveness can be the model of EPC intervention (monoprofessional vs. multiprofessional; remote vs in-person specialized contact), risk of contamination and/or crossover with the conventional arm, study duration, timing of evaluation, timeliness of cohort inception (this will probably differ for different cancer types), level of symptom burden, and reduced QoL of recruited patients (11).

A topic under discussion is whether palliative care can be provided by professionals with two different levels of expertise: a basic one (for example, oncologists capable of ensuring a good palliative approach) and a specialized one (palliative care specialists who are dedicated full-time to palliative care delivery). These two levels are indeed both essential, and one of them alone cannot exhaustively perform the functions of both (12–14). The specification of hematological pathologies could lead to the identification of specific models of EPC intervention.

Finally, it is possible that different health systems, in low-income and high-income countries, recognize the need for different models of PC integration (6).

From what is described above, it follows that some aspects relating to EPC have been confirmed, while for others further research is necessary. The process of collating articles on these topics aimed to highlight issues still worthy of research that had not been completely clarified.

Some articles in this Special Issue have focused on the most appropriate way to activate EPC. The integration of clinical practice and education programs and clinical research can demonstrate the usefulness of PC programs in cancer research centers and promote program penetrance. (Alquati et al.) Moreover, a qualitative study reports how improved communication by treating oncologists can contribute to a timely and appropriate activation of EPC (Collins et al.).

The search for triggers to integrate standardized early palliative care (STEP care) has proved to be feasible for certain primary neoplasms (brain) but not for others, representing the need to activate EPC in a modulated way for individual pathologies and not in the same way for all tumors, binding to specific objectives, and identifiable and visible parameters (Collins et al.). A very early screening of palliative care needs can be performed in low-income countries with a simple and reproducible instrument such as the Distress Thermometer (Abu-Odah et al.). Even in developed countries, a structured low-threshold screening program for supportive and palliative can preserve several dimensions of quality of life as a comprehensive multidimensional assessment (Solar et al.).

Some outcomes of EPC were also evaluated. It has been reported that therapeutic appropriateness can occur in palliative radiotherapy using validated prognostic scores, including the Palliative Prognostic Score (PaP Score) already used in other end-of-life settings (Maltoni et al.). Another proved outcome is gratitude from patients and their families for the care received in the EPC program (Borelli et al.). Finally, particular attention should be paid to the outcomes of the EPCs relating to sub-populations that require particular attention and support: women and younger adults (Galiano et al.).

Finally, two papers focus on the specifics of early palliative care in hematological and neurological pathologies (Tanzi and Martucci, Armitage and Fonkem).

The paper on PC research in hematologic cancer patients provides an expert opinion about what works, and how and for whom, in facilitating enrollment in PC studies for patients with hematological malignancies. A qualitative review regarding contexts, mechanisms, and outcomes (CMOs) was performed, and the resulting theory was informed by narrative research, along with a structured interview of PC experts and a pilot study by the authors. The work identifies some crucial points to carry out PC research in hematology, concerning the mutual perceptions of the different actors and the relationships between PC specialists and hematologists (Tanzi and Martucci).

The article concerning neurodegenerative pathologies underlines the aggressiveness of some neurological tumors compared to other non-oncological neurodegenerative pathologies slowly progressing. Glioblastoma (WHO Grade IV) and Parkinson’s disease are mentioned as archetypes of the two trends. As result of a qualitative review of the literature, summarized by expert opinions, in glioblastoma the timely referral to early palliative care is recommended to improve quality of life, ensure a dignified death, and alleviate and ease grief and burden for family members and other caregivers. Specific points that are discussed are diagnosis, progression, and prognostication; patient and family education; and caregiver burden (Armitage and Fonkem).

In conclusion, medical oncology and palliative care are mutually necessary, and early and progressive integration must be pursued, with the support of clinical and organizational, which must be systematically and continuously promoted and supported (15–17).

## Author contributions

Conception and final approval: All authors. Manuscript writing: MM.
